# ZnO-Based Microcavities Sculpted by Focus Ion Beam Milling

**DOI:** 10.1186/s11671-016-1526-2

**Published:** 2016-06-30

**Authors:** Tsu-Chi Chang, Kuo-Bin Hong, Ying-Yu Lai, Yu-Hsun Chou, Shing-Chung Wang, Tien-Chang Lu

**Affiliations:** Department of Photonics, National Chiao Tung University, 1001 University Road, Hsinchu, 300 Taiwan

**Keywords:** ZnO, Microcavity, Microdisk, Whispering gallery mode, Focus ion beam

## Abstract

We reported an easy fabrication method to realize ZnO-based microcavities with various cavity shapes by focused ion beam (FIB) milling. The optical characteristics of different shaped microcavities have been systematically carried out and analyzed. Through comprehensive studies of cathodoluminescence and photoluminescence spectra, the whispering gallery mode (WGM) was observed in different shaped microcavities. Up further increasing excitation, the lasing action was dominated by these WGMs and matched very well to the simulated results. Our experiment shows that ZnO microcavities with different shapes can be made with high quality by FIB milling for specific applications of microlight sources and optical devices.

## Background

Recently, micro/nanoscience made a great progress and attracted extensive research efforts because they have potential applications in optoelectronic devices, such as microlight sources, photo-switches, and optical integrated circuits [1–4]. ZnO is considered to be one of the promising materials for making microsize devices, which would be able to operate at ultraviolet (UV) region due to its wide bandgap of about 3.37 eV and large exciton binding energy of about 60 meV at room temperature [5]. In addition, the specific crystal facets of single crystalline wurtzite ZnO bulk parallel to the c-plane have a naturally hexagonal cross section, which would be able to readily serve as a high-quality whispering-gallery mode (WGM) resonator owing to its relatively high reflective index (~2.4) in comparison to the surrounding air. The high-quality factor (Q) of WGM microcavity (MC) could be achieved by the total internal reflection (TIR) that could facilitate to further reduce the lasing threshold. Over the past decades, the ZnO-based WGM optical resonator was first reported by Nobis et al. [6] and the corresponding WGM lasing action was observed in a ZnO nanonail [7].

In terms of fabrication of ZnO MCs, less studies utilized top-down etching technique [8, 9] because it might require complex fabrication steps as well as subsequent precise patterning procedure. In addition, it was difficult to define the sample position and the material quality was limited by the substrate. Therefore, the fabrication of low dimensional laser resonators by top-down approach remains a challenging task. In contrast, bottom-up synthesized nanostructures that usually form hexagonal symmetry of crystal morphology have inherent advantages over top-down fabricated structures such as high material quality, smooth facet, and high assembly throughput. ZnO nano/microstructures have a perfect hexagonal cross section, and WGM in such hexagonal structure has been experimentally studied in detail in ZnO micro- and nanowires and disks [4, 7, 10–12]. Light propagating around the WGM resonator due to the total internal reflection effect has been investigated by cathodoluminescence (CL) [10, 13, 14] and photoluminescence (PL) [2, 11]. Various bottom-up fabrication methods have been reported to realize ZnO nanostructures, such as hydrothermal [3, 4], chemical vapor deposition (CVD) [11, 12, 15], and vapor phase transport (VPT) [2, 13, 14] method. However, the challenge of bottom-up synthesized ZnO MC is the controllability in position of a single microcavity because the spatial isolation is important for optically investigating one single nanoscale cavity [2]. The spatial distribution of samples made by the bottom-up synthesis method usually appears clustering arrangement. To study optical characterization of an individual object could be tedious process. Recently, many researchers have demonstrated the capability to fabricate various types of polygonal microcavities, but the morphology and size of the microcavities are difficult to control [9, 12, 16–18]. On the other hand, the top-down approach could benefit from the ready single crystalline ZnO material that could provide very good optical characteristics and laser gain. However, the ZnO-based single crystalline resonators are rarely reported because of the difficult fabrication process, which strongly depends on the availability of substrate [15, 19].

Recently, we have successfully fabricated the membrane-type ZnO MC. The ZnO membrane was cut from a single crystalline ZnO substrate by using focused ion beam (FIB) milling. However, this fabrication process only allows us to realize a square-shaped ZnO MC [20]. In this work, we developed an easy method to fabricate ZnO MCs with a controllable submicrometer spatial resolution to realize various shape ZnO MCs in which whispering-gallery mode lasing can be achieved. To obtain a high-quality MC, the starting material was the ZnO bulk substrate. Then, the FIB milling and glass tip technique were applied for the cavity formation on the ZnO bulk substrate and position to the targeted substrate. The narrow linewidth WGM mode lasing was observed in circular, hexagonal, pentagonal, and square resonators, verified by using the microphotoluminescence (μ-PL) system. Detailed characteristics of whispering-gallery mode lasing microcavities have been discussed and analyzed.

## Methods

**Fig. 1:**
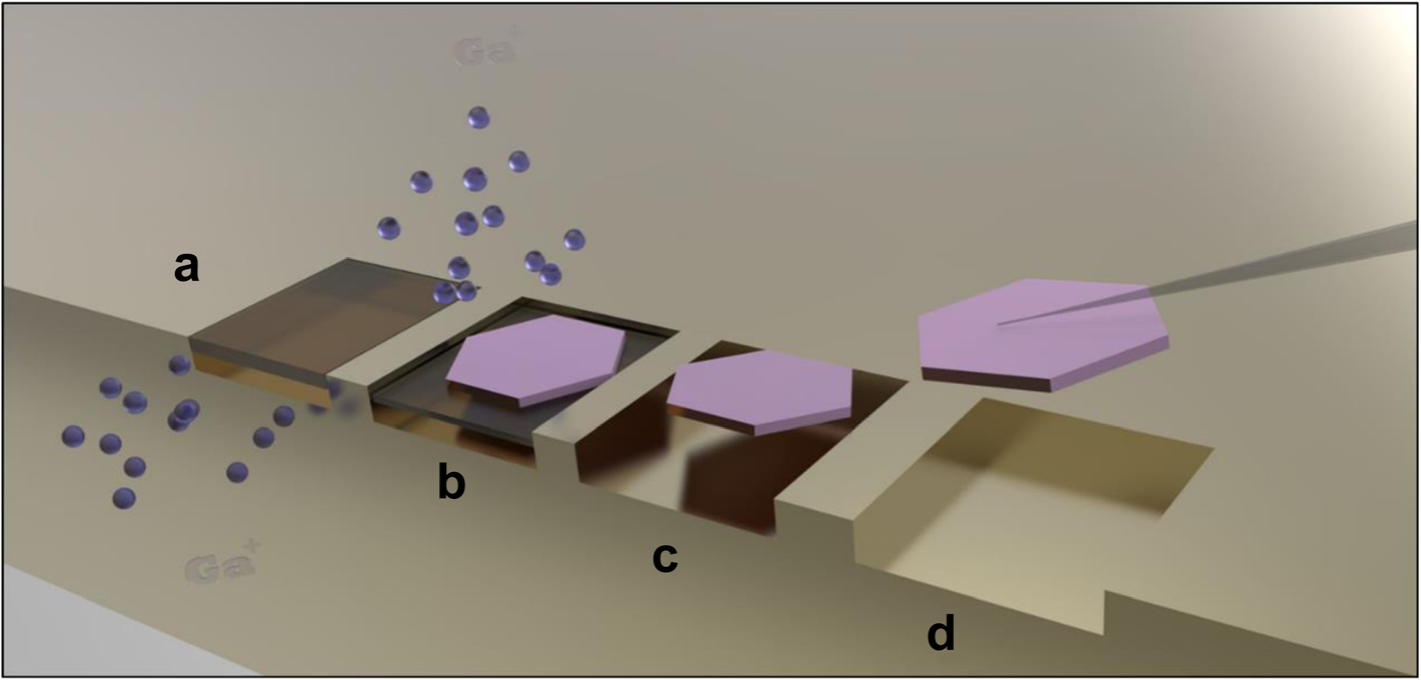
Schematic illustration of single crystalline ZnO MC fabrication process. **a** The thin film was formed by carving into the ZnO substrate using conventional top-down FIB etching. **b** The pattern was defined and etched by standard ion beam milling. **c** FIB etching was further employed to realize the suspended ZnO MC. **d** The ZnO MC was picked up by using a glass tip

**Fig 2:**
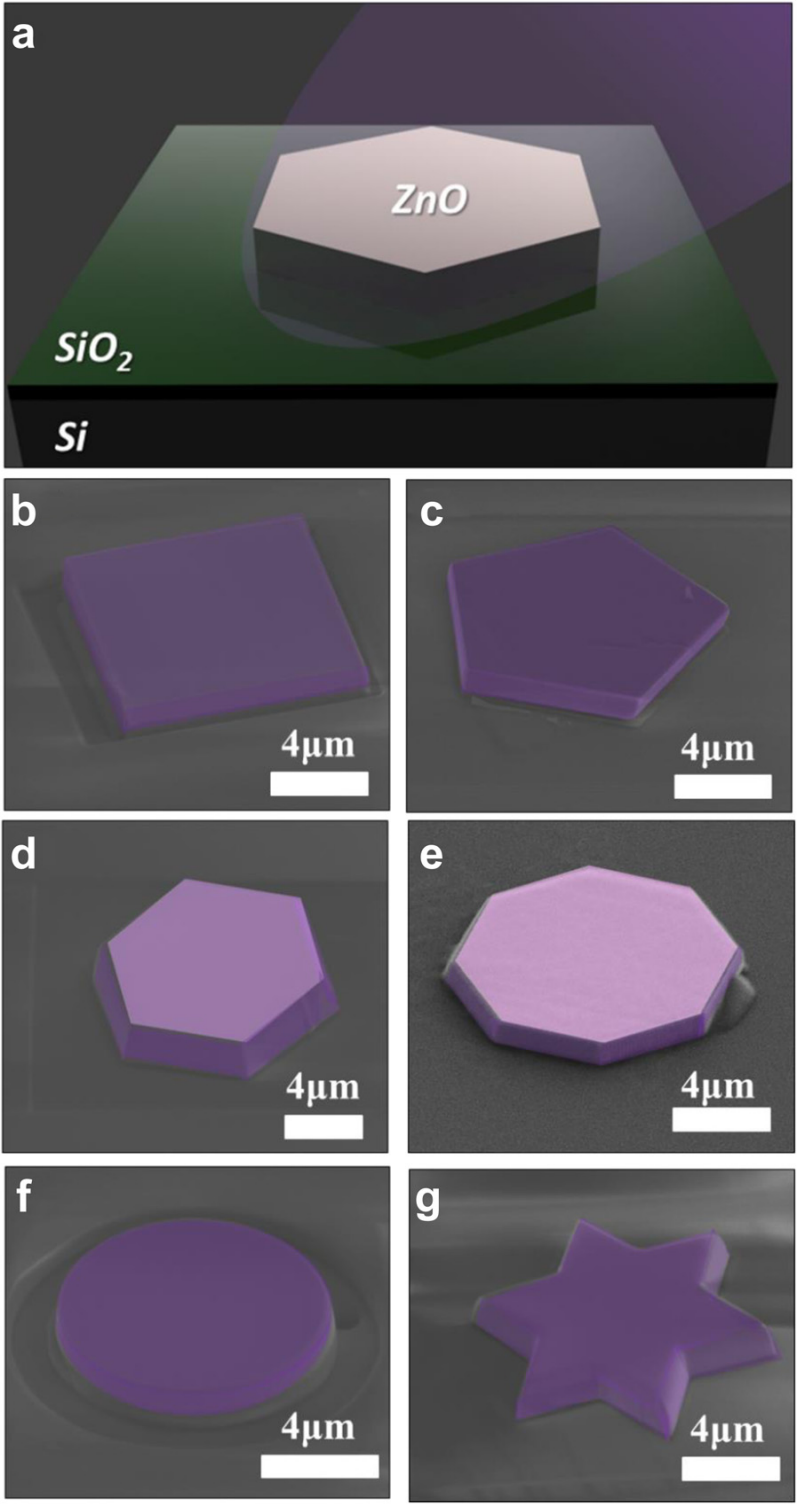
**a** The illustration of a ZnO MC placed on the SiO_2_/Si substrate for optical measurement. SEM images of ZnO MCs with different shapes including the **b** square, **c** pentagon, **d** hexagon, **e** octagon, **f** circle, and **g** hexagram

The single crystalline c-plane ZnO bulk substrate was used as the starting materials for making microcavities. Figure 1 illustrates the simple FIB etching process flow for carving the ZnO microdisks with different shapes. The FIB milling provides a quick and easy way to obtain a better quality thin film sample with an arbitrary shape. The fabrication process is described as followed. First, the edge side of the ZnO bulk was lifted up, and a conventional top-down FIB etching was used to dig a hollow for forming the suspended thin film as shown in Fig. 1a. The uniform thickness was the key in this step. The fabrication was performed using a dual beam system (focused ion beam and electron beam). The thickness of ZnO film was controlled by an oblique etching and immediately measured in the electron beam system to monitor the thickness uniformity. The thickness and area of thin film were about 1 μm and 10 × 10 μm^2^, respectively. Then, we can engrave the ZnO thin film into any desired shape, as illustrated in Fig. 1b. Next, the sample was placed down to let the suspended thin film faced up so that the suspended ZnO thin film could be sculpted by the FIB milling with a slower etching rate for fabricating the defined shape MCs, as shown in Fig. 1c. Finally, the ZnO MC was picked up and placed onto the SiO_2_/Si substrate by the use of a glass tip, as illustrated in Figs. 1d and 2a.

Figure 2a is a schematic of the ZnO MC put on the SiO_2_/Si substrate. The 2-μm-thick SiO_2_ was grown by the wet oxidation method, and this SiO_2_ layer was used to serve as a low index layer to properly support the high Q WGM. The ZnO MC was excited by the 355-nm third-harmonic generation of an Nd:YVO_4_ pulse laser with 0.5-ns duration and 1-kHz repetition rate, and the pumping spot size was approximately 30 μm. The resultant fluorescence emitting from the ZnO MC was collected through an optical microscope with an objective lens of ×100 and then coupled to a spectrometer through an optical fiber. Figure 2b to g displays the scanning electron microscopy (SEM) images of suspended ZnO MCs with square, pentagon, hexagon, octagon, circle, and even hexagram shapes, fabricated by the FIB technique. Specifically, the various shaped ZnO MCs shown in Fig. 2 were realized with a perfect symmetry, which were considered to be difficult by using aforementioned conventional fabrication methods. From these SEM images, the sidewalls of ZnO MCs have smooth facets that are benefit to form the WGM resonance. Therefore, we have clearly demonstrated symmetric or even exotic ZnO MCs which can be realized by this technique.

## Results and Discussion

**Fig 3:**
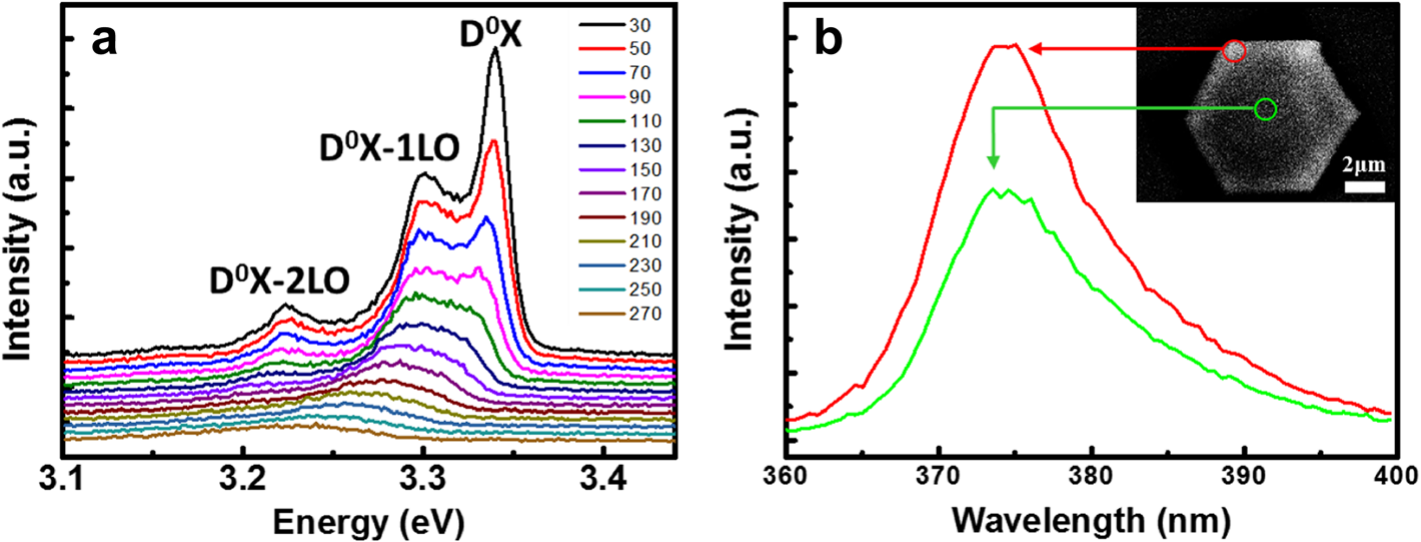
**a** Temperature-dependent CL spectra of a ZnO MC. **b** Monochromatic CL image of the ZnO MC. Representative CL spectra recorded from the side region and the center region of the ZnO MC. The local excitation is indicated by *red* and *green circles* in monochromatic CL image. *Inset* of **b** is the lateral spread of the interaction volume of the electron beam at 15 kV with a scale bar of 2 μm. There is a significant difference of emission between the side and the center regions

CL measurement of the samples was carried out at 30 to 270 K with an accelerating voltage of 15 kV inside a SEM. The temperature-dependent CL spectra are shown in Fig. 3a. Typically, the exciton of the wurtzite ZnO existed in various forms, which can be classified into free and bound excitons (FX and BX). The free excitons usually only appear in high material quality samples. Basically, the BX energy is lower than FX, which can also be classified into donor bound exciton (DX) and acceptor bound exciton (AX). During the optical process of ZnO, the strong Fröhlich interaction between electrical filed of longitudinal optical (LO) phonon and the dipole moment of excitons makes it easy to release LO phonons and form several LO phonon replicas. Therefore, at 30 K, the dominant peak is seen at 3.34 eV, which can be attributed to the FX emission (denoted as D_0_X) and its first- and second-order LO phonon replicas separated uniformly by 75.6 meV [3, 15, 21]. That indicates ZnO LO phonons were experienced very efficient coupling between excitons and phonons, demonstrating that our fabricated ZnO MCs possessed very good crystal quality. The monochromatic CL image and representative spectra of hexagonal ZnO MC at 374 nm is shown in Fig. 3b. As can be seen in the image, the bright luminescence is locally concentrated at the boundary of hexagonal MC. The CL spectra show the emission from the side and center regions, respectively. Both CL spectra have intensity peak at 374 nm but the luminescence emission was much stronger at the boundary of the MC, indicating that the total internal reflection of light could occur at the side walls of the MC.

**Fig 4:**
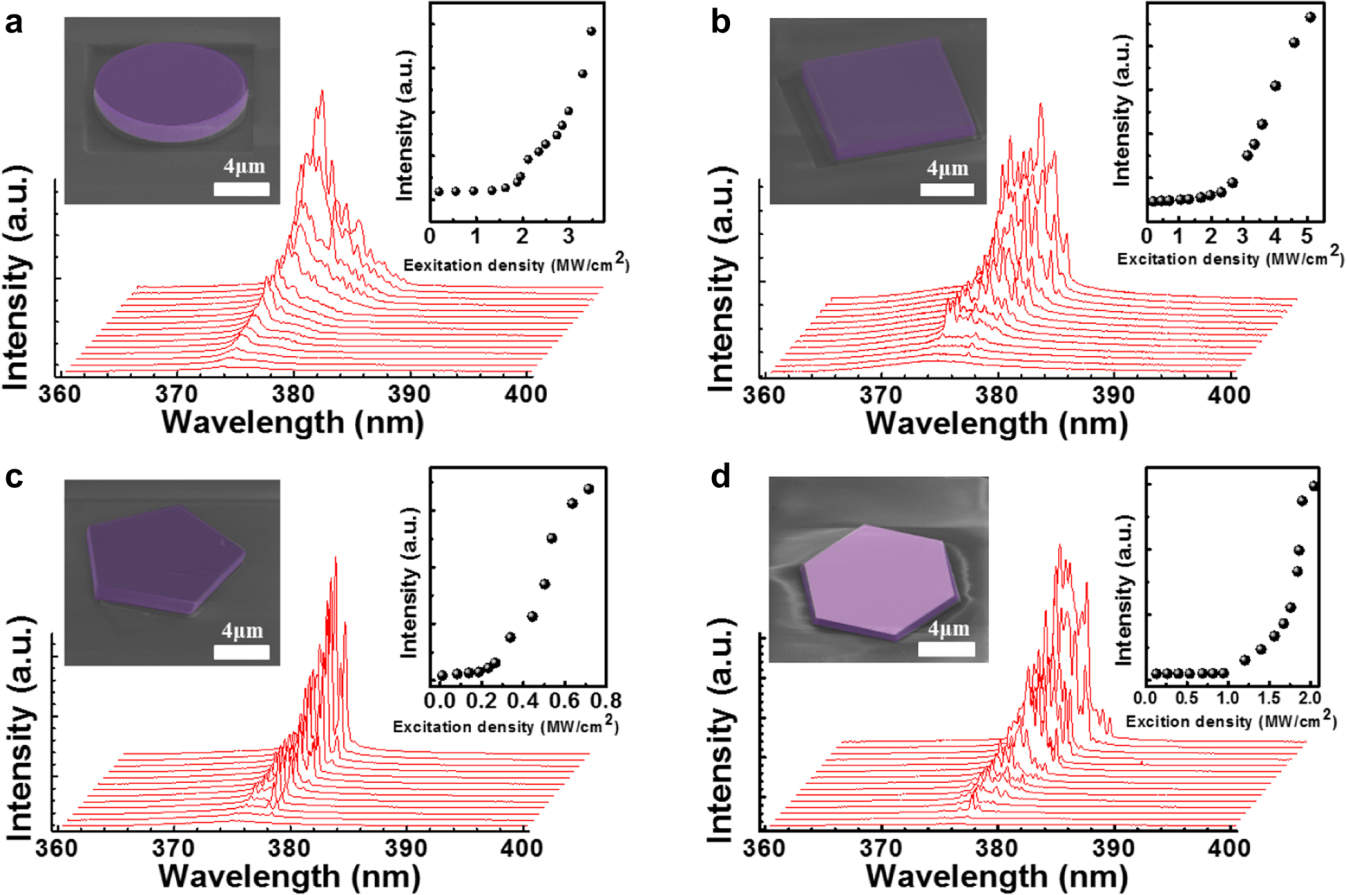
Power-dependent lasing spectra of ZnO MCs with **a** circular, **b** square, **c** pentagonal, and **d** hexagonal shapes. *Red lines* represent distinct emission spectra under different pumping power densities. *Insets* in the figures show the integrated PL intensity as a function of excitation energy density labeled by *black solid dots*. The threshold power densities are 1.94, 2.63, 0.26, and 1.33 MW/cm^2^ for circular, square, pentagonal, and hexagonal shapes, respectively

To demonstrate the capability of achieving the WGM lasing of ZnO MCs, we fabricated four types of ZnO MCs including circular, square, pentagonal, and hexagonal shapes employed for further optical characterization. Figure 4 shows the emission spectra of ZnO MCs under different pumping intensities, while the insets show the SEM images of the MCs with different shapes and the integrated PL intensity as a function of the excitation density, respectively. The integrated PL intensities show nonlinear increasing and second slopes as the excitation density reaches the threshold with sharp multiple-peak spectra, indicating that all four samples indeed achieved laser action. The threshold power densities of circle, square, pentagon, and hexagonal were 1.94, 2.63, 0.26, and 1.33 MW/cm^2^, respectively. For the hexagonal MC, the ZnO disks reported in the previous references were all fabricated by using vapor phase transport (VPT) and the corresponding laser threshold was 0.28MW/cm^−2^ [19]. Moreover, the laser threshold of our hexagonal sample is very similar to that obtained for VPT. Interestingly, a relatively low lasing threshold of 0.26 MW/cm^2^ was observed in the pentagon MC compared with other shaped MCs. Accordingly, it was also worth noting that narrower spectral line width and fewer lasing modes were observed in the pentagon-shaped ZnO MC. The reason why the pentagon-shaped ZnO MC exhibited a lower threshold power than other samples will be discussed subsequently.

Inside a ZnO MC, WGM can be formed by bouncing near the ZnO/air interfaces due to the relatively large refractive index contrast to sustain the total internal reflection (TIR). In order to verify the WGM characteristics from different shaped ZnO MCs, theoretical calculations of WGM mode numbers were performed. The wavelength-dependent refractive index of ZnO crystals measured at 77 K were taken from the previous report and fitted with the Sellmeier’s formula [22]. The fitted dispersion relation of ZnO used in this paper can be expressed as follows:

1 $$ \mathrm{T}\mathrm{E}:\kern1em {n}_{\mathrm{TE}}\left(\uplambda \right)={\left(1+\frac{2.026{\lambda}^2}{\lambda^2-{263.796}^2}+\frac{0.063{\lambda}^2}{\lambda^2-{366.778}^2}+\frac{0.025{\lambda}^2}{\lambda^2-{11485.8}^2}\right)}^{1/2} $$

2 $$ \mathrm{T}\mathrm{M}:\kern0.24em {n}_{\mathrm{TM}}\left(\uplambda \right)={\left(1+\frac{2.234{\lambda}^2}{\lambda^2-{243.974}^2}+\frac{0.082{\lambda}^2}{\lambda^2-{361.675}^2}+\frac{0.013{\lambda}^2}{\lambda^2-{11259.9}^2}\right)}^{1/2} $$

Taking into account the WGM mode numbers for each peak wavelength observed from PL measurements, we deduced the following mode number equations for regular circle, square, pentagon, and hexagon cavities through a classical plane-wave mode for the WGM cavity of different shapes [23]. The integer *N* in the Eqs. (2)–(5) denotes the mode number of transverse-electric-polarized (TE) WGMs, *D* is the side length of cavity, and the index *n* can be obtained from Eq. (1).

3 $$ \mathrm{W}\mathrm{G}\mathrm{M}\ \mathrm{f}\mathrm{o}\mathrm{r}\ \mathrm{circle}\ \mathrm{M}\mathrm{C}:N=\frac{nD\pi }{\lambda } $$

4 $$ \mathrm{W}\mathrm{G}\mathrm{M}\ \mathrm{f}\mathrm{o}\mathrm{r}\ \mathrm{square}\ \mathrm{M}\mathrm{C}:N=\frac{2\sqrt{2}nD}{\lambda }-\frac{\pi }{4}{ \tan}^{-1}\left(n\sqrt{n^2-2}\right) $$

5 $$ \mathrm{W}\mathrm{G}\mathrm{M}\ \mathrm{f}\mathrm{o}\mathrm{r}\ \mathrm{pentagon}\ \mathrm{M}\mathrm{C}:N=\frac{5\sqrt{1+2/\sqrt{5}}nD}{\sqrt{2/\sqrt{5}+2}\lambda }-\frac{\pi }{5}{ \tan}^{-1}\left(n\sqrt{\frac{\left(5-\sqrt{5}\right){n}^2-8}{2+\sqrt{5}}}\right) $$

6 $$ \mathrm{W}\mathrm{G}\mathrm{M}\ \mathrm{f}\mathrm{o}\mathrm{r}\ \mathrm{hexagon}\ \mathrm{M}\mathrm{C}:N=\frac{3\sqrt{3}nD}{\lambda }-\frac{\pi }{6}{ \tan}^{-1}\left(n\sqrt{3{n}^2-4}\right) $$

7 $$ \mathrm{Quasi}\hbox{-} \mathrm{W}\mathrm{G}\mathrm{M}\ \mathrm{f}\mathrm{o}\mathrm{r}\ \mathrm{hexagon}\ \mathrm{M}\mathrm{C}:N=\frac{9nD}{\lambda }-\frac{\pi }{3}{ \tan}^{-1}\left(n\sqrt{\frac{\left({n}^2-4\right)}{3}}\right) $$

The transverse-magnetic-polarized (TM) WGMs can also be obtained in a similar way. For the ZnO MC that contains c-plane facets, the WGM modes mainly occur in the c-plane of ZnO which is parallel to the normal surface of as-prepared samples because the TE fields in the c-plane of ZnO exhibits a greater optical gain than the TM-polarized emission [24]. However, in some cases, the TM-polarized WGMs could be observed if the proper excitation is applied on the MCs.

**Fig 5:**
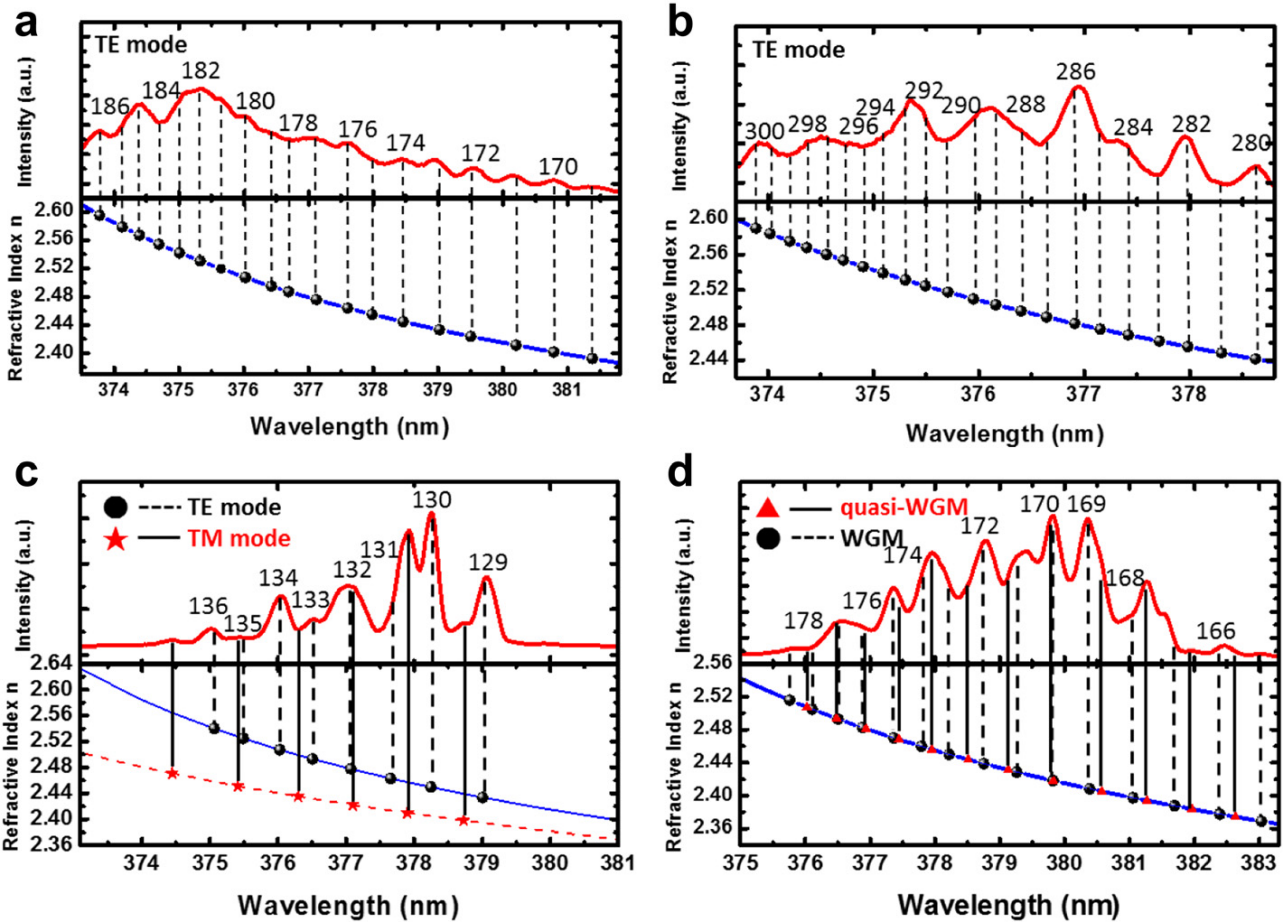
The WGM mode numbers for the lasing spectra and corresponding refractive indices for **a** circular, **b** square, **c** pentagonal TE and TM mode, and **d** quasi-WGM and WGM in hexagonal shape ZnO MCs. Numbers in the subfigures are mode numbers of WGM calculated from the by Eqs. (1)-(5)

The WGM mode numbers associated with PL peaks of circular-, square-, pentagonal-, and hexagonal-shaped ZnO MCs, and corresponding refractive indices are shown in Fig. 5. As it can be seen, most of the measured PL lasing peaks follow closely with the calculated WGM with dispersive refractive indices. The circle, square and hexagon ZnO MCs showed densely distributed TE-polarized WGM peaks in the lasing spectra, which followed the previous description on the larger optical gain for TE polarization in the c-plane ZnO MCs. In addition, the hexagon ZnO MC demonstrated quasi-WGM peaks in the lasing spectra, indicating a highly symmetric morphology of the fabricated MC.

However, among four samples, the pentagon-shaped MC exhibited less resonant peaks with low-order resonant modes. This is because the pentagon does not belong to the symmetric group of polygon so that fewer allowed round-trip paths could be found. Therefore, the TM-polarized lasing peaks were observed due to the lesser mode competition for the same laser gain. Similarly, the decrease in the lasing threshold of pentagonal ZnO MC could be attributed to the fewer WGMs presented in the PL spectra which further mitigated the presence of mode competition. The laser mode competition can be simply explained by the following expression by using the linear gain approximation [25].

8 $$ {n}_{th}=\frac{N_c}{a{\tau}_p{\upsilon}_g}+{n}_{tr} $$

where *N*_*c*_ is the number of allowed optical modes within the gain bandwidth, *n*_*th*_ is the threshold carrier density, *a* is the differential gain, υ_g_ is the group velocity, τ_p_ is the photon lifetime, and *n*_*tr*_ is the transparency carrier density. It can be seen that the threshold carrier density would be increased as more lasing modes participating the same exciton reservoir. In contrast, the limited cavity mode numbers in the pentagon ZnO MC actually resulted in a lower threshold power.

**Fig 6:**
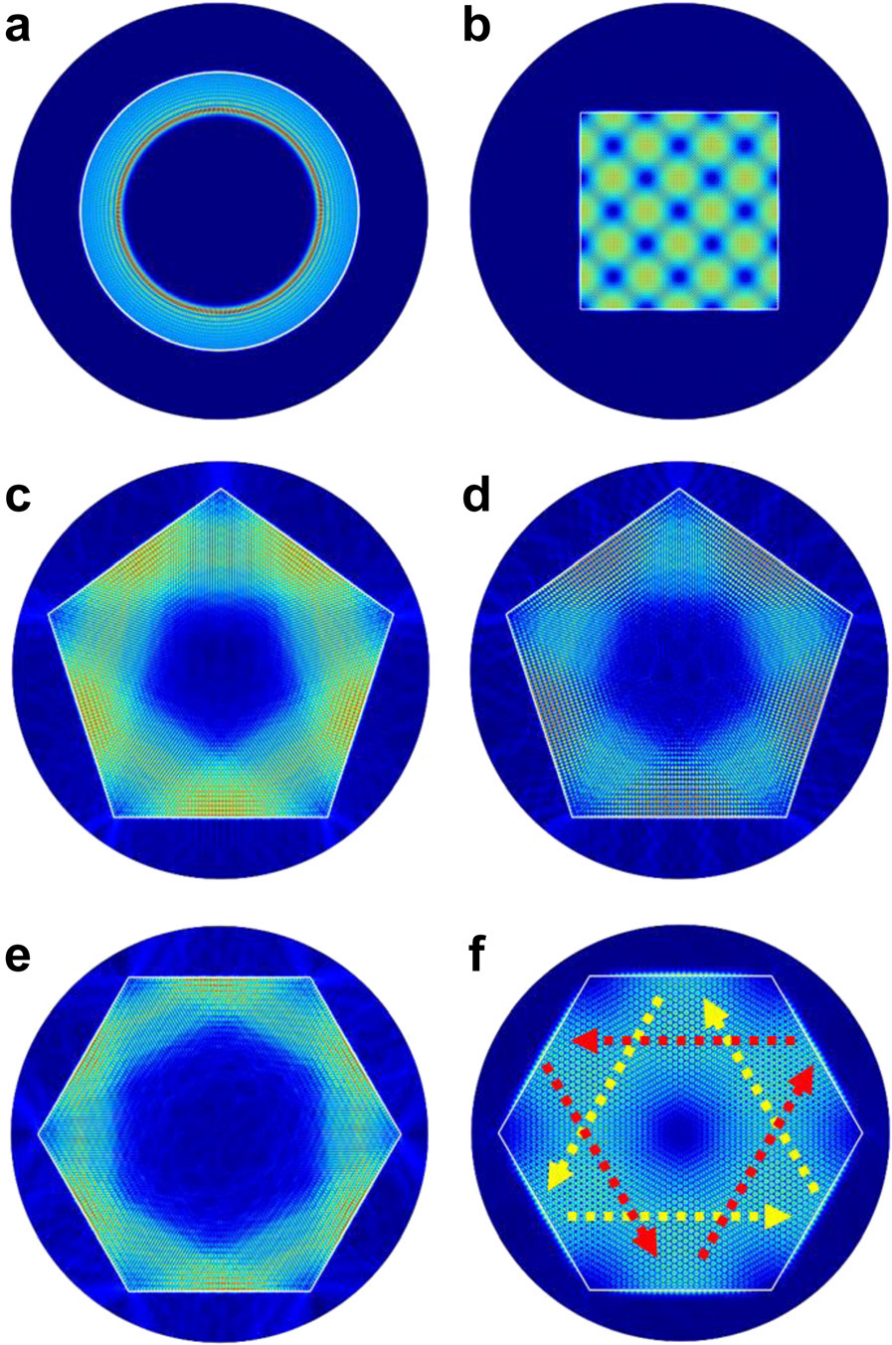
Simulated electric field profiles of WGM modes for ZnO MCs with **a** circular, **b** square, **c**, **d** pentagonal TE and TM mode, and **e**, **f** hexagonal WGM and quasi-WGM mode for mode wavelength at 377.19, 377.05, 375.71, 375.46, 379.82, and 379.83 nm, respectively. *White lines* indicated the boundaries of ZnO MCs

Furthermore, we used the finite element approach to calculate the WGM by using the commercial software to see the electric field profiles of the ZnO MCs with circular, square, pentagonal, and hexagonal shapes, as shown in Fig. 6. It can be clearly seen that the bright fringes show the eigenmodes traveling across the cavity. Figure 6a, b shows the complicated optical mode distribution in the cavity. These resonant modes can be attributed to the TE-polarized WGMs in circle and square ZnO MCs. In the hexagon MC, light path can be hexagonal and triangular (indicated by dashed arrows) as normal and quasi WGM shown in Fig. 6e, f. In the pentagon MC, we showed both TE and TM modes in Fig. 6c, d. It is interesting to note that Fig. 6c, d shows good overlap between the excitation power and the tracing path of WGMs in the pentagon MC. Together with less resonant modes involved in the laser gain bandwidth, lower threshold pumping power density can be obtained in the pentagon MC.

## Conclusions

We presented a novel bulk nanomachining technique for carving various polygonal MCs. The different shapes of single crystalline ZnO MCs were realized with circle, square, pentagon, hexagon, octagon, and even hexagram shapes. The fabrication process is achieved by FIB milling and subsequent utilizing a glass tip to control the exact position of samples. The lasing characteristics of circular, square, pentagonal, and hexagonal MCs were further measured and analyzed. The experimental results showed the pentagon cavity has potential to achieve WGM lasing with the lowest threshold power density of 0.26 MW/cm^2^ owing to the cavity exhibiting limited resonant modes. Our study revealed that the FIB milling could be a handy process to design and fabricate different kinds of MCs and the inherent good crystal properties could be simultaneously preserved for further practical coherent microlight sources in integrated photonic devices and optical biosensor applications.
